# Bleeding, thrombosis, and anticoagulation in myeloproliferative neoplasms (MPN): analysis from the German SAL-MPN-registry

**DOI:** 10.1186/s13045-016-0242-9

**Published:** 2016-03-05

**Authors:** A. Kaifie, M. Kirschner, D. Wolf, C. Maintz, M. Hänel, N. Gattermann, E. Gökkurt, U. Platzbecker, W. Hollburg, J. R. Göthert, S. Parmentier, F. Lang, R. Hansen, S. Isfort, K. Schmitt, E. Jost, H. Serve, G. Ehninger, W. E. Berdel, T. H. Brümmendorf, S. Koschmieder, for the Study Alliance Leukemia (SAL)

**Affiliations:** Department of Hematology, Oncology, Hemostaseology, and Stem Cell Transplantation, Faculty of Medicine, RWTH Aachen University, Pauwelsstr. 30, 52074 Aachen, Germany; Internal Medicine 3, Oncology, Hematology and Rheumatology, University Clinic Bonn (UKB), Bonn, Germany; Practice for Hematology and Oncology, Wuerselen, Germany; Department for Hematology, Oncology, Stem Cell Transplantation, Hospital Chemnitz, Chemnitz, Germany; Department for Hematology, Oncology and Clinical Immunology, University Hospital Duesseldorf, Duesseldorf, Germany; Practice for Hematology-Oncology Eppendorf, Hamburg, Germany; Department for Hematology, University Hospital Dresden, Dresden, Germany; Practice for Hematology and Oncology Altona, Hamburg, Germany; Department for Hematology, University Hospital Essen, Essen, Germany; Department for Hematology, Oncology and Palliative Care, Rems-Murr-Hospitals, Winnenden, Germany; Department for Hematology and Oncology, University Hospital Frankfurt/Main, Frankfurt/Main, Germany; Practice for Hematology and Oncology, Kaiserslautern, Germany; Department of Medicine A, University Hospital Münster, Münster, Germany

**Keywords:** MPN, PV, ET, PMF, MPN-U, Thrombosis, Thromboembolism, Major bleeding, Anticoagulation

## Abstract

**Background:**

Patients with Ph-negative myeloproliferative neoplasms (MPN), such as polycythemia vera (PV), essential thrombocythemia (ET), and primary myelofibrosis (PMF), are at increased risk for thrombosis/thromboembolism and major bleeding. Due to the morbidity and mortality of these events, antiplatelet and/or anticoagulant agents are commonly employed as primary and/or secondary prophylaxis. On the other hand, disease-related bleeding complications (i.e., from esophageal varices) are common in patients with MPN. This analysis was performed to define the frequency of such events, identify risk factors, and assess antiplatelet/anticoagulant therapy in a cohort of patients with MPN.

**Methods:**

The MPN registry of the Study Alliance Leukemia is a non-interventional prospective study including adult patients with an MPN according to WHO criteria (2008). For statistical analysis, descriptive methods and tests for significant differences as well as contingency tables were used to identify the odds of potential risk factors for vascular events.

**Results:**

MPN subgroups significantly differed in sex distribution, age at diagnosis, blood counts, LDH levels, JAK2V617F positivity, and spleen size (length). While most thromboembolic events occurred around the time of MPN diagnosis, one third of these events occurred after that date. Splanchnic vein thrombosis was most frequent in post-PV-MF and MPN-U patients. The chance of developing a thromboembolic event was significantly elevated if patients suffered from post-PV-MF (OR 3.43; 95 % CI = 1.39–8.48) and splenomegaly (OR 1.76; 95 % CI = 1.15–2.71). Significant odds for major bleeding were previous thromboembolic events (OR = 2.71; 95 % CI = 1.36–5.40), splenomegaly (OR = 2.22; 95 % CI 1.01–4.89), and the administration of heparin (OR = 5.64; 95 % CI = 1.84–17.34). Major bleeding episodes were significantly less frequent in ET patients compared to other MPN subgroups.

**Conclusions:**

Together, this report on an unselected “real-world” cohort of German MPN patients reveals important data on the prevalence, diagnosis, and treatment of thromboembolic and major bleeding complications of MPN.

## Background

Philadelphia-chromosome (Ph-neg) negative myeloproliferative neoplasms (MPN) are a heterogeneous group of rare hematopoietic stem cell clonal diseases. According to the WHO 2008 classification, Ph-neg MPN include classical MPN, such as essential thrombocythemia (ET), polycythemia vera (PV), and primary myelofibrosis (PMF), as well as less common entities such as chronic neutrophilic leukemia (CNL), hypereosinophilic syndrome (HES), systemic mastocytosis (SM), and unclassifiable MPN (MPN-U) [1]. Various recurrent molecular alterations have been described in classical MPN, such as JAK2 V617F [2], MPL W515L/K [3], or MPL S505 mutations and deletion or insertions in the calreticulin gene. In addition, further mutations in other genes, such as CBL, CHEK2, DNMT3A, ASXL1, EZH2, IDH1/2, SF3B1, SH2B3 (LNK), SETBP1, SRSF2, and TET2, have been found in MPN [4–7].

These genetic factors at least in part play a causal role in the disease pathogenesis of MPN and have greatly facilitated the diagnostic work-up [8].

MPN are known to be associated with an increased risk of thrombotic and thromboembolic events compared to the general population, and these events contribute considerably to morbidity and mortality of MPN [9–14]. On the other hand, MPN patients are also at a higher risk for bleeding complications due to antiplatelet and anticoagulant therapy necessary to prevent major thromboembolic complications in high-risk patients. Another important risk factor for bleeding complications is the presence of esophageal or gastric varices due to portal vein hypertension and/or an acquired von Willebrand syndrome (AVWS) due to excessive thrombocytosis [15–18].

During the last decade, novel insights into the pathogenesis and the risk factors of thromboembolic events in MPN have been gained. Apart from known thrombosis-associated risk factors such as a previous thrombotic event or age of the patient, typical MPN-associated risk factors have also been described [19]. Endothelial activation, polyglobulia (PV), and leukocyte activation [20–22] are among the most relevant risk factors, while divergent results were reported regarding high platelet counts and thrombosis [9, 23]. Notably, JAK2 positivity is also a strong risk factor for vascular events when compared to JAK2 Ph-negative MPN. In contrast, MPL and calreticulin mutations are not associated with an increased risk of thrombosis [8, 9, 24, 25].

Regarding bleeding risk, disease-related as well as therapy-associated factors have to be considered, including anticoagulation with vitamin K antagonists (VKA), novel anticoagulatory drugs (NOAC), and antiplatelet therapy [26] but also AVWS [27] and storage pool defects with a downregulation of glycoprotein (GP)Ib and GPIIb/IIIa [28]. The role of an imbalance of distinct platelet surface receptors is still debated, since platelet surface receptor expression also differs significantly in “healthy” subjects [29]. However, there is evidence for a direct effect of JAK2 V617F, which has been documented to influence platelet activation in ET via a complex mechanism of the PI3K/Rap1 pathway leading to impaired thrombopoietin-mediated integrin IIb 3 activation [30]. Furthermore, hypersplenism and thrombocytopenia may also enhance the risk of bleeding, especially in MF patients.

This analysis of data from the MPN registry of the German Study Alliance Leukemia (SAL) was performed to describe, in a “real-world” setting of German MPN patient care, the incidence of thrombotic and thromboembolic as well as major bleeding events before, at the time of diagnosis and during clinical follow-up of Ph-negative MPN patients with a particular focus on the underlying diagnosis, treatment modality, and patient-related factors potentially affecting vascular events in MPN. The ultimate goal of this study was to elucidate vascular and bleeding complications in a representative group of patients with Ph-negative MPN and to identify potential risk factors for the development of these events.

## Results

**Fig. 1:**
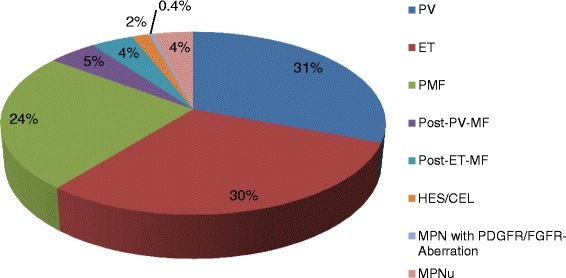
Distribution of the MPN subtypes in the registry (*n* = 466). Classical MPN, PV, ET, and PMF represent 85 % of all subtypes, followed by post-PV and post-ET myelofibrosis and MPN-U. Documentation of CNL, HES/CEL, SM, and MPN with a PDGFR-alpha, PDGFR-beta, or FGFR1-aberration was infrequent (together 2.4 % of all subtypes)

Patients’ general characteristics are shown in Table 1. Among the MPN subtypes, significant differences were observed for gender, JAK2 V617F status, spleen size, and relevant laboratory parameters. Figure 1 shows the distribution of the MPN subtypes included in this MPN registry. Ninety-four percent of all cases were so-called classical MPN, such as PV, ET, and primary and secondary myelofibrosis, while 4 % were MPN-U cases.

**Table 1:** General characteristics of all patients with MPN (*n* = 455)

	All patients	PV	ET	PMF	Post-PV-MF	Post-ET-MF	MPN-U	*p* value
Patients; *n* (% of total)	454	142 (31.3)	140 (30.8)	113 (24.9)	22 (4.8)	19 (4.2)	18 (4.0)	
Male sex; *n* (%)	232 (51.1) *n* = 454	65 (45.8) *n* = 142	57 (40.7) *n* = 140	76 (67.3) *n* =113	11 (50) *n* = 22	12 (63.2) *n* = 19	11 (61.1) *n* = 18	0.0007^#^
Age at diagnosis; median, mean (SD)	60 57.7 (15.2) *n* = 442	60 59.2 (13.9) *n* = 140	54 52.9 (16.5) *n* = 137	61.5 60.6 (13.4) *n* = 108	63 58.4 (12.6) *n* = 21	68 63.9 (17.8) *n* = 18	57.5 57 (18.4) *n* = 18	0.0008*
Hematocrit^a^; median, mean (SD)	42.5 41.9 (10.9) *n* = 441	50 49.5 (10.6) *n* = 139	41.6 40.4 (7.9) *n* = 137	34.6 35.4 (9.6) *n* = 108	46 42.7 (10) *n* = 21	34.7 34.7 (7.9) *n* = 18	42.1 38.9 (7.2) *n* = 18	<0.0001*
Platelets^a^; median, mean (SD)	516 575.2 (383.7) *n* = 441	487 522.2 (269.3) *n* = 137	704 789.1 (410.1) *n* = 139	376 429.4 (374.4) *n* = 109	395.5 446.2 (325.8) *n* = 20	279.5 359.3 (271.1) *n* = 18	502.5 568.3 (425.3) *n* = 18	<0.0001*
LDH^a^; median, mean (SD)	307 415.2 (332.5) *n* = 409	282 308.3 (121.2) *n* = 128	252 314.2 (209.9) *n* = 130	462 565.8 (326.8) *n* = 101	593.5 717.9 (599.5) *n* = 18	670 913.7 (835.8) *n* = 15	310 336.8 (162) *n* = 17	<0.0001*
Jak2V617F-positive; *n* (%)	289 (75.5) *n* = 383	108 (91.5) *n* = 118	71 (61.7) *n* = 115	68 (68) *n* = 100	18 (100) *n* = 18	9 (60) *n* = 15	15 (88.2) *n* = 17	<0.0001^#^
Spleen in cm by ultrasound^a^; median, mean (SD)	14.5 15.6 (5.4) *n* = 243	13.8 14.6 (3.7) *n* = 75	12.5 13.2 (3.4) *n* = 61	16.2 16.5 (4.2) *n* = 69	23.1 23.9 (12.7) *n* = 14	17.4 18.3 (5.3) *n* = 14	15.5 16.1 (3.9) *n* = 10	<0.0001*

*Wilcoxon-Mann-Whitney test; ^#^Chi-square test

^a^At the time of the first admission

Of all patients, 33.6 % suffered from a vascular event. The most frequent events were deep vein thrombosis (31.5 %), acute coronary syndrome (27.7 %), stroke (19.3 %), and splanchnic vein thrombosis (15.2 %). For splanchnic vein thrombosis, a significant difference between the MPN subtypes was detected (*p* = 0.0083) (Table 2), as it was most frequent in MPN-U (60 %), followed by post-PV MF (30.8 %).

**Table 2:** Thrombosis/thromboembolism and bleeding events in MPN

	All Pts	PV	ET	PMF	Post-PV-MF	Post-ET-MF	MPN-U	*p* value^#^
Thrombosis/thromboembolism^a^; *n* (%)	147 (33.6) *n* = 438	54 (38.9) *n* = 139	33 (25) *n* = 132	34 (31.2) *n* = 109	13 (61.9) *n* = 21	8 (42.1) *n* = 19	5 (27.8) *n* = 18	*0.0120*
DVT; *n* (%)	46 (31.5) *n* = 146	15 (27.8) *n* = 54	10 (30.3) *n* = 33	11 (33.3) *n* = 33	4 (30.8) *n* = 13	3 (37.5) *n* = 8	3 (60) *n* = 5	0.7781
ACS; *n* (%)	41 (27.7) *n* = 148	15 (27.8) *n* = 54	12 (35.3) *n* = 34	7 (20.6) *n* = 34	4 (30.8) *n* = 13	2 (25) *n* = 8	1 (20) *n* = 5	0.8439
Stroke; *n* (%)	28 (19.3) *n* = 145	13 (24.5) *n* = 53	7 (21.2) *n* = 33	7 (21.2) *n* = 33	0 (0) *n* = 13	0 (0) *n* = 8	1 (20) *n* = 5	0.2714
SVT; *n* (%)	22 (15.2) *n* = 145	3 (5.7) *n* = 53	6 (18.2) *n* = 33	6 (18.2) *n* = 33	4 (30.8) *n* = 13	0 (0) *n* = 8	3 (60) *n* = 5	*0.0083*
Bleeding^a^, *n* (%)	36 (8.2) *n* = 437	13 (9.4) *n* = 139	5 (3.8) *n* = 133	10 (9.3) *n* = 108	4 (19.1) *n* = 21	1 (5.3) *n* = 19	3 (17.7) *n* = 17	0.0586
Upper GI-Tract-Bleeding; *n* (%)	20 (55.6) *n* = 36	7 (53.9) *n* = 13	3 (69) *n* = 5	6 (60) *n* = 10	3 (75) *n* = 4	0 (0) *n* = 1	1 (33.3) *n* = 3	0.8748
Postinterventional; *n* (%)	4 (11.1) *n* = 36	3 (23.1) *n* = 13	0 (0) *n* = 5	0 (0) *n* = 10	0 (0) *n* = 4	1 (100) *n* = 1	0 (0) *n* = 3	0.0958
CNS; *n* (%)	3 (8.3) *n* = 36	0 (0) *n* = 13	0 (0) *n* = 5	3 (30) *n* = 10	0 (0) *n* = 4	0 (0) *n* = 1	0 (0) *n* = 3	0.1725

*Pts* patients, *DVT* deep vein thrombosis, *ACS* acute coronary syndrome, *SVT* splanchnic vein thrombosis, *CNS* central nervous system

^#^In Fisher’s exact test

^a^Life-time events

Major bleeding events were reported in 8.2 % of all patients, with upper gastrointestinal bleedings being most common (55.6 % of all bleeding occurrences), and other bleeding causes being significantly less frequent. As shown in Table 2, we detected significant differences in the proportion of major bleeding between the different MPN subtypes.

**Fig. 2:**
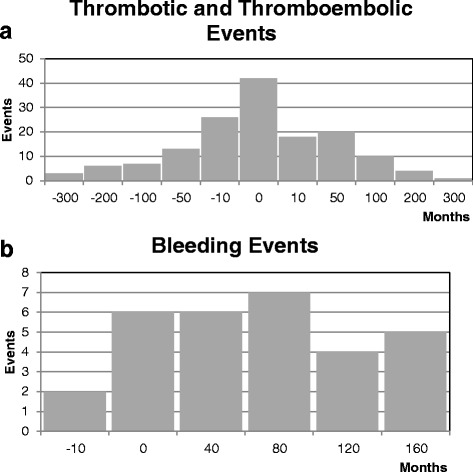
Number of thrombotic/thromboembolic (**a**) and major bleeding events (**b**) in MPN over time in months. The *0* (zero) marks the date of diagnosis. In **a** (thrombotic and thromboembolic events), there are several events before and after diagnosis which presents almost like a normal distribution. While in **b** (bleedings events), there were only two cases of major bleedings described before date of diagnosis—all other major bleedings occurred after that date

Figure 2 shows the number of thrombotic/thromboembolic and bleeding events over time in relation to the date of diagnosis (time point “zero”). For vascular occlusions, the distribution of events was similar before and after MPN diagnosis (Fig. 2 a). In contrast, hemorrhages were only rarely detected before diagnosis, as only two out of 36 events occurred prior to diagnosis, whereas all other bleeding events occurred after MPN had been diagnosed (Fig. 2b). Bleeding may be supported by antiplatelet and anticoagulant therapy; thus, we next evaluated their association with bleeding episodes.

In the overall cohort, antiplatelet and anticoagulant therapy was frequently applied. Most patients were exposed to acetylsalicylic acid (ASS) (57.4 % of all patients) and oral VKA (almost 10 %). Of note, eight patients (1.8 %) received the NOAC rivaroxaban.

With regard to the MPN-specific therapy, watchful waiting strategies were most frequently applied (51.7 %), followed by the use of hydroxyurea (49.2 %) as cytoreductive therapy (Table 3). Based on the long clinical course, patients received different substances and/or therapy regimes during their follow-up. In 87 patients (19.9 %), the JAK1/2 inhibitor ruxolitinib was administered, most frequently in MF patients (Table 3).

**Table 3:** Antiplatelet and anticoagulant therapy and anti-MPN therapy

	All patients	PV	ET	PMF	Post-PV-MF	Post-ET-MF	MPN-U	*p* value^#^
Antiplatelet drugs								
ASS^a^; *n* (%)	248 (57.4) *n* = 432	92 (67.2) *n* = 137	83 (61.5) *n* = 135	45 (42.9) *n* = 105	11 (61.1) *n* = 18	10 (52.6) *n* = 19	7 (38.9) *n* = 18	*0.0026*
P2Y12 antagonist^a^; *n* (%)	27 (6.4) *n* = 425	9 (6.7) *n* = 134	13 (9.7) *n* = 134	4 (3.9) *n* = 104	0 (0) *n* = 17	0 (0) *n* = 18	1 (5.6) *n* = 18	0.4064
Anticoagulants								
VKA; *n* (%)	43 (9.8) *n* = 437	14 (10.1) *n* = 138	13 (9.5) *n* = 137	6 (5.6) *n* = 107	4 (21.1) *n* = 19	3 (16.7) *n* = 18	3 (16.7) *n* = 18	0.1511
Rivaroxaban; *n* (%)	8 (1.8) *n* = 437	3 (2.2) *n* = 138	1 (0.7) *n* = 137	4 (3.7) *n* = 107	0 (0) *n* = 19	0 (0) *n* = 18	0 (0) *n* = 18	0.6151
Heparin; *n* (%)	16 (3.8) *n* = 417	4 (3.1) *n* = 131	4 (3.1) *n* = 131	5 (4.9) *n* = 103	2 (11.1) *n* = 18	1 (5.9) *n* = 17	0 (0) *n* = 17	0.4345
Anti-MPN therapy^b^								
Watch wait; *n* (%)	226^c^ (51.7) *n* = 437	79 (57.7) *n* = 137	72 (52.9) *n* = 136	49 (46.2) *n* = 106	7 (33.3) *n* = 21	6 (31.6) *n* = 19	13 (72.2) *n* = 18	*0.0321*
Hydroxyurea; *n* (%)	215 (49.2) *n* = 437	82 (59.9) *n* = 137	67 (49.3) *n* = 136	38 (35.9) *n* = 106	10 (47.6) *n* = 21	9 (47.4) *n* = 19	9 (50) *n* = 18	*0.0151*
Anagrelide; *n* (%)	66 (15.1) *n* = 437	15 (11.0) *n* = 137	32 (23.5) *n* = 136	11 (10.4) *n* = 106	3 (14.3) *n* = 21	4 (21.1) *n* = 19	1 (5.6) *n* = 18	*0.0255*
Ruxolitinib; *n* (%)	87 (19.9) *n* = 437	15 (11.0) *n* = 137	5 (3.7) *n* = 136	43 (40.6) *n* = 106	11 (52.4) *n* = 21	12 (63.2) *n* = 19	1 (5.6) *n* = 18	*<0.0001*
IMIDe; *n* (%)	21 (4.8) *n* = 437	2 (1.5) *n* = 137	0 (0) *n* = 136	15 (14.2) *n* = 106	2 (9.5) *n* = 21	1 (5.3) *n* = 19	1 (5.6) *n* = 18	*<0.0001*
IFNα; *n* (%)	40 (9.2) *n* = 437	12 (8.8) *n* = 137	15 (11.0) *n* = 136	9 (8.5) *n* = 106	2 (9.5) *n* = 21	1 (5.3) *n* = 19	1 (5.6) *n* = 18	0.9681
SCT; *n* (%)	20 (4.6) *n* = 439	3 (2.2) *n* = 139	0 (0) *n* = 134	12 (11.1) *n* = 108	2 (9.5) *n* = 21	2 (10.5) *n* = 19	1 (5.6) *n* = 18	*<0.0001*

*VKA* vitamin K antagonist, *WatchWait* watchful waiting, *SCT* stem cell transplantation

^#^In Fisher’s exact test

^a^Double platelet inhibition in *n* = 20 patients (4.72 %)

^b^Life-time therapy

^c^ *n* = 114 patients (26 %) solely had watchful waiting strategy

As expected, the type of MPN therapy differed significantly among the MPN subtypes (Table 3), except for interferon alpha (n.s. most likely due to small numbers of patients receiving this agent).

Tables 4 and 5 show the odds for the development of thrombotic/thromboembolic and major bleeding events. Significantly elevated odds ratios for the occurrence of thromboembolism were found for a diagnosis of post-PV-MF and splenomegaly, but not the other subtypes, JAK2 V617F, leukocytosis, or thrombocytosis (Table 4). Significantly elevated odds ratios for major bleeding events were found for those patients with a history of a thrombotic or thromboembolic event, splenomegaly, and those with a therapy with heparin. The administration of a P2Y12 antagonist or double platelet inhibition showed a positive trend for the latter (Table 5). Neither thrombocytosis nor thrombocytopenia nor the use of ASS, VKA, or NOAC was associated with a significant odds ratio for major bleeding events. Intriguingly, in our cohort, the diagnosis of ET was associated with a significantly reduced odds ratio for major bleeding events as compared to the other MPN subgroups, with a trend towards higher bleeding frequencies in post-PV-MF and MPN-U patients. Furthermore, the diagnosis ET showed a protective effect for thrombotic/thromboembolic events (OR = 0.56; 95 % CI 0.36–0.89). A subgroup analysis of patients with PMF, post-PV-MF, or post-ET-MF showed that in all of these subgroups, a low platelet count might be a mild risk factor for major bleeding events but this did not reach statistical significance (data not shown). In a logistic regression model for the prediction of bleeding events, the variables age, splenomegaly, thrombotic/thromboembolic event in medical history, administration of ASS, P2Y12 antagonist, heparin, VKA, and rivaroxaban were stepwise selected to enter and remain in the model. Only the variable “thrombotic/thromboembolic event in medical history” showed a significant effect (*p* = 0.0032).

**Table 4:** Odds ratios for thromboembolism

	Odds ratio (OR)	95 % CI
Diagnosis		
PV	1.4309	0.9398–2.1786
ET	*0.5614*	*0.3554–0.8867*
PMF	0.8665	0.5445–1.3792
Post-PV-MF	*3.4319*	*1.3892–8.4783*
Post-ET-MF	1.4650	0.5762–3.7248
MPN-U	0.7530	0.2632–2.1540
Jak2V617F-positive	1.4785	0.8732–2.5034
High leukocytes (>25/nl)	1.1500	0.6593–2.0059
High platelets (>1000/nl)	0.7163	0.3738–1.3727
Splenomegaly (detected by palpation)	*1.7623*	*1.1480–2.7052*

**Table 5:** Odds ratios for major bleeding events

	Odds ratio (OR)	95 % CI
Diagnosis		
PV	1.2480	0.6123–2.5439
ET	*0.3440*	*0.1307–0.9053*
PMF	1.1892	0.5539–2.5531
Post-PV-MF	2.8235	0.8964–8.8935
Post-ET-MF	0.6079	0.0788–4.6904
MPN-U	2.5130	0.6872–9.1897
Thrombotic/thromboembolic event in medical history	*2.7083*	*1.3578–5.4021*
Splenomegaly (detected by palpation)	*2.2222*	*1.0095–4.8919*
Low platelets (<100/nl)	1.3120	0.5504–3.1275
High platelets (>1000/nl)	1.1874	0.4401–3.2035
ASS	1.1216	0.5539–2.2712
P2Y12 antagonist	2.8292	0.9979–8.0213
Double platelet inhibition	3.0500	0.9589–9.7016
Oral vitamin K antagonist	1.9739	0.7695–5.0634
Rivaroxaban	1.6092	0.1923–13.4665
Heparin	*5.6426*	*1.8360–17.3421*

Significant results are in italics

For a multivariate logistic regression model (including age class, splenomegaly, Jak2 status, high leukocytes, high platelets, cancer) to predict thrombosis/thromboembolism in our cohort, only splenomegaly was detected to have a significant effect on the prediction of thrombosis/thromboembolism (*p* = 0.0009 in maximum likelihood test).

## Discussion

Arterial and venous thrombosis/thromboembolism significantly contributes to morbidity and mortality of MPN patients [18, 31]. We here describe the frequency and risk factors for thrombotic/thromboembolic and major bleeding events in a cross section of patients with classical MPN and MPN-U in Germany, using clinical data from the SAL-MPN registry. In contrast to many clinical trials, our “real-world” analyses include a largely unselected group of MPN patients, ranging from newly diagnosed patients to those with a long disease history and covering different health care service settings (university hospitals, non-university hospitals, and office-based physicians) mirroring organization of hematological patient care in the German health care system.

Approximately 50 % of analyzed patients were male which is in accordance with recently published data [32], whereas the median age at first diagnosis was considerably lower (57 years) in our study. This difference may be caused by a selection bias (selected MPN centers documentation vs population-wide cancer registry) and the time period of inclusion (2012–2015 vs 1980–2009). Alternatively, the difference may be due to an earlier diagnosis in today’s patients.

About one third of the evaluated patients suffered from arterial or venous thromboembolism. DVT was the most common event followed by cardiac events, which is in accordance with previous findings [31, 33]. Regarding the subtype analysis, especially in PV and ET, the occurrence of thromboembolic complications was similar to previous findings [31, 34]. Interestingly, only in approximately one quarter of patients developing a thrombosis/thromboembolism subsequent events were registered, suggesting a lower frequency than in the published literature (33.6 % [26]) and, possibly, a more effective cytoreduction, anticoagulation, or antiplatelet therapy after the initial event.

Atypical venous thrombosis occurs more frequently in MPN patients, when compared to patients without MPN [35, 36]. We also report splanchnic vein thrombosis (SVT) accounting for 15 % of all thromboembolic events. However, in our series, the proportion of SVT in PV and ET was lower (2–5 % in all patients) than previously reported in the literature (10–13 %) [34], and PV patients had less SVT events compared to other MPN subtypes, which also reached statistical significance in our cohort. Others support our observation that SVT is less common in PV compared to ET [31], whereas other studies did not find significant differences in the different MPN subgroups. Differences among the studies, including our own, may be caused by a reporting bias or the small numbers of events per group (e.g., only three events in the PV group).

Patients from our cohort were less likely to develop a recurrent thrombosis, with only one fifth of patients in our cohort suffering from two or more thromboembolic events, and this proportion was clearly lower than in other studies (about 30 %) [26]. This may be due to a lower MPN-dependent and MPN-independent risk of thrombosis/thromboembolism, as evidenced by the younger age of the patients in our cohort and/or the high fraction of patients that were followed by watchful waiting.

Our univariate analyses revealed that only post-PV-MF diagnosis and splenomegaly were significant risk factors for the development of a thrombotic/thromboembolic event. In contrast to reports from the literature, a high white blood count (WBC) and JAK2V617F positivity were no identifiable risk factors for such events in our study [9, 16, 21, 24, 26].

Thrombotic/thromboembolic events are crucial factors of morbidity and mortality in PV and ET [10, 14, 37]. The particular time point of the events is meaningful, notably to detect vulnerable phases in the clinical course. In our cohort, most patients’ thrombotic/thromboembolic events peaked around the time of diagnosis, with an almost normal distribution around this time point. This suggests that thrombotic/thromboembolic events constitute a major indicator of an MPN and often triggers MPN diagnosis. However, it also suggests that some patients may have thrombotic/thromboembolic events already long before the diagnosis of MPN. This should be taken into account when strategies of enhancing public awareness of MPN and prevention of thrombotic/thromboembolic complications are concerned. Furthermore, the distribution of events may be an indicator for successful strategies in preventing recurrent thrombotic/thromboembolic events and suggest that a rigorous work-up regarding a potential underlying MPN should be initiated, particularly in patients with SVT.

Strikingly, the distribution of major bleedings followed a different pattern, without a peak around diagnosis but rather with most events occurring after the diagnosis of an MPN. This suggests that major bleeding occurs as a consequence of an MPN itself (i.e., ET-associated AVWS), portal hypertension with esophageal varices due to MPN-associated SVT, or primary prophylactic MPN therapy (e.g., ASS in PV) or anticoagulation in patients with previous thrombosis [27–29].

In our cohort, the overall major bleeding rate was 8 % which is close to the rates described in other studies [26, 38, 39]. However, while nearly 10 % of PV patients had a major hemorrhage, which is significantly higher than in the published literature [38], major bleeding rated in our ET patients were slightly below the published data [38]. The reduced frequency of bleedings in ET patients could be due to a higher proportion of patients receiving cytoreduction as well as the restrictive use of antiplatelet therapy with regard to an acquired von Willebrand Syndrome (AVWS), similar to what has been described for the ANAHYDRET trial [39]. Furthermore, the OR of 0.34 as a protective effect of bleeding events corroborates a careful and optimized therapy of ET patients documented in the SAL-MPN registry. Though the ET diagnosis also leads to a protective effect for thrombotic/thromboembolic events, an insufficient treatment with antiplatelet or anticoagulative substances cannot be assumed.

Current clinical guidelines recommend the administration of ASS for all PV patients to prevent thrombotic/thromboembolic events [31]. However, only about two third of PV patients in our series received ASS. Contraindications such as gastric ulcers and esophageal varices were detected in 16 patients. Other reasons for the lack of ASS administration could be the administration of anticoagulant therapy (e.g., VKA), as the combination of VKA and antiplatelet therapy should be only used with caution [31]. Our data further illustrate that so far, only a minority of patients with MPN received NOAC. And the risk of bleeding cannot currently be adequately assessed. However, with the development of the dabigatran antidote idarucizumab, there are new therapeutic options in case of major bleeding occurring in dagibatran-treated patients [40].

In the analysis of potential risk factors for major bleeding events, a previous history of vascular events, splenomegaly, and the administration of heparin were identified. Interestingly, neither ASS nor VKA were identified as risk factors. This lacking association, especially for patients receiving VKA, may be due to an intense surveillance of this cohort. P2Y12 antagonists as well as double-agent antiplatelet therapy narrowly missed the significance level, possibly due to small sample size (27 and 20 patients). These findings, especially the elevated odds of developing a major bleeding with heparin, need to be evaluated in future prospective studies. Although bleeding events are not the main cause for mortality in MPN patients [41], the prevention of such incidents is crucial, especially in case of long-term antiplatelet and anticoagulative treatment [42]. Clinical recommendations frequently discuss this topic and develop strategies to prevent bleeding caused by the antithrombotic medication [12, 17, 18, 31, 38].

In the past years, survival of MPN patients, especially for PV and ET subtypes has improved [13], yet the relative 5-year survival for MPN patients in Germany decreases from 92.3 % at ages 14–49 years to 63 % at 70+ years of age [43]. Reasons for the higher mortality rates in elderly patients were, in particular, thrombotic/thromboembolic events [10, 14, 44]. Our cohort showed a low proportion of recurrent thrombosis but a high frequency of thrombosis after the date of diagnosis. Intriguingly, 10 PV patients and 6 ET patients from our cohort had a thrombotic/thromboembolic event but did not receive cytoreductive therapy. Besides cytoreductive therapy for high-risk PV and ET, control of cardiovascular risk factors (smoking, dyslipidemia, hypertension) is crucial and should not be neglected [10, 18, 21, 37, 45, 46].

We would like to discuss our findings with regard to the already existing European LeukemiaNet (ELN) guidelines [47] and guidelines of the German and Austrian Society of Hematology and Oncology (DGHO/ÖGHO) and the Society of Thrombosis and Haemostasis Research (GTH) [31]. According to ELN guidelines, antiplatelet therapy with ASS in PV patients is recommended for all patients unless there is a contraindication. ASS was not shown to increase the bleeding risk in this patient cohort. For ET patients, ASS is recommended in case of microvascular disturbances according to ELN guidelines. In all patients “aspirin should be withdrawn in the event of major bleeding, most frequently GI, or in the rare cases of allergy or intolerance” [47]. Our own results, presented in this study, confirm the safety of aspirin in this patient cohort. But we suggest to restrict the use of aspirin in patients without a clear indication. Furthermore, the ELN consortium gave a detailed recommendation on SVT management. “Treatment of splanchnic vein thrombosis includes low molecular weight heparin followed by [life-long] oral anticoagulation […]” [47]. However, the ELN recommendations did not contain further detailed recommendations on the management of other thrombotic/thromboembolic.

In our study, the administration of vitamin K antagonists (VKA) did not result in an increased major bleeding risk, which is in accordance to the findings of the DGHO/ÖGHO/GTH recommendations paper [31]. Conversely, our study suggests that the administration of P2Y12 antagonists might be associated with major bleeding events. However, further evaluations are needed, in light of the DGHO/ÖGHO/GTH guidelines currently recommending the administration of P2Y12 antagonists in case of ASS allergy or intolerance [31].

Interestingly, in the RESPONSE trial, which assessed the JAK inhibitor ruxolitinib vs standard therapy for the treatment of polycythemia vera, a significant reduction of thromboembolic events was seen in the ruxolitinib group [48]. Since ruxolitinib is known to reduce spleen size, we investigated whether enlargement of the spleen is a risk factor for thrombotic/thromboembolic events. Indeed, our study showed that splenomegaly was a risk factor for thrombotic/thromboembolic events in our cohort. Potentially, a reduction of the spleen size in MPN patients may be an attractive future goal to reduce the incidence of thromboembolic events.

Several limitations of our study should be acknowledged. As mentioned above, patients’ data were obtained from MPN centers in Germany and not from a country-wide cancer registry, which may be an explanation for the lower median age of first diagnosis. Therefore, we cannot exclude a selection bias, since the centers that participate in our registry have a special interest in MPN pathogenesis and treatment, rendering generalization of the results difficult. Additionally, no data regarding length, intensity, and combination of any specific or general treatments was gathered. Furthermore, our cohort was very broad regarding the time of data collection. Some patients entered the registry at the time of diagnosis and others after a long latency period. It is imaginable that, in particular for patients with longstanding MPN history, thrombotic/thromboembolic, or bleeding events may have been underreported if they occurred before referral to the participating centers. The number of patients and the low incidence of these diseases (1–3 per 100 000 inhabitants) is also a limiting factor of this study and might affect our results.

On the other hand, the strength of our registry is the inclusion of patient data from academic centers, community hospitals, or office-based environments, providing a representative picture of MPN-care in Germany. Thus, the reported patients much more reflect the “real-world” population without the plethora or restrictings generated by stringent in-/exclusion criteria of patients treated in controlled clinical trials.

## Conclusions

In summary, thrombotic/thromboembolic and bleeding events play an important role in the clinical course of MPN patients. However, one third of all thrombotic/thromboembolic events in our registry occurred after diagnosis, although it is known that these events mainly lead to morbidity and mortality in MPN patients, and standard treatment of PV patients (i.e., with ASS) did not always reflect current clinical guidelines. Thus, it will be important to address these points in the future observation and interventional clinical trials. On the other hand, ET patients showed a reduced occurrence of bleeding complications which may be an indicator that the risk factors for major hemorrhages in these patients are well appreciated in the clinical practice nowadays and that current therapy concepts appropriately address these risk factors.

## Methods

### Patients and clinical data

The German SAL-MPN registry is a national prospective observational study with several university hospitals, community hospitals, and hematology/oncology practices participating in the documentation of patients with MPN. Inclusion criteria were the following: a confirmed MPN diagnosis according to the WHO classification (2008) or IWG-MRT criteria, patient age of 18 years or older, and written informed consent by the patient. The MPN registry is approved by the Ethics Committee of the Medical Faculty of RWTH Aachen University (EK 127/12) as well as each local Ethics Committees of the participating centers. Patient recruitment started in August 2012, the here presented contain clinical data from 454 patients with PV, ET, PMF, post-PV-MF, post-ET-MF, and MPN-U that were available for statistical analyses until data lock in February 2015.

Clinical data include laboratory results, molecular genetics, clinical signs and symptoms, and complications such as vascular or major bleeding events (defined as intracranial or retroperitoneal bleed or associated with a decrease in hemoglobin ≥2 g/dl or requiring of blood transfusions [38]) as well as concomitant medication and MPN-specific therapy.

### Statistical analysis

Clinical data was collected and analyzed using SAS Software (SAS 9.3, SAS Institute Inc., Cary, NC, USA). First, descriptive analyses of general characteristics, thrombotic/thromboembolic, and major bleeding events as well as concomitant medication/procedures and therapies were performed for characterization of the MPN cohort. Chi-square test, Fisher’ exact test, and Wilcoxon-Mann-Whitney test were used to describe the distribution of categorical and continuous variables between the different subtypes. Contingency tables were used to identify the odds of potential risk factors for thrombotic/thromboembolic and major bleeding events. A logistic regression model was generated for the prediction of bleeding and vascular events in MPN. In this analysis, a stepwise selection of defined variables was performed with a significance of level of *p* = 0.10 to enter and a *p* value of 0.05 to remain in the model.

All statistical tests were two-sided, and *p* < 0.05 was used as the level of significance.
